# Dynamic Capture and Release of Endoplasmic Reticulum Exit Sites by Golgi Stacks in Arabidopsis

**DOI:** 10.1016/j.isci.2020.101265

**Published:** 2020-06-12

**Authors:** Junpei Takagi, Yoshitaka Kimori, Tomoo Shimada, Ikuko Hara-Nishimura

**Affiliations:** 1 Faculty of Science and Engineering, Konan University, Kobe 658-8501, Japan; 2 Faculty of Environmental and Information Sciences, Fukui University of Technology, Fukui 910-8505, Japan; 3 Graduate School of Science, Kyoto University, Kyoto 606-8502, Japan

**Keywords:** Biological Sciences, Cell Biology, Plant Biology

## Abstract

Protein transport from the endoplasmic reticulum (ER) to Golgi stacks is mediated by the coat protein complex COPII, which is assembled at an ER subdomain called ER exit site (ERES). However, the dynamic relationship between ERESs and Golgi stacks is unknown. Here, we propose a dynamic capture-and-release model of ERESs by Golgi stacks in *Arabidopsis thaliana*. Using variable-angle epifluorescence microscopy with high-temporal-resolution imaging, COPII-component-bound ERESs were detected as punctate structures with sizes of 300–500 nm. Some punctate ERESs are distributed on ER tubules and sheet rims, whereas others gather around a Golgi stack in an ER-network cavity to form a beaded-ring structure. Free ERESs that wander into an ER cavity are captured by a Golgi stack in a cytoskeleton-independent manner. Then, they are released by the Golgi stack for recycling. The dynamic ERES cycling might contribute to efficient transfer of *de novo* synthesized cargo proteins from the ER to Golgi stacks.

## Highlights

• VAEM images show dynamic behavior of minimal punctate ERESs
• Most of punctate ERESs are distributed on the ER network tubes
• Several punctate ERESs contact with a Golgi stack in an ER network cavity
• ERESs are dynamically captured and released by Golgi stacks

## Graphical Abstract

**Figure:**
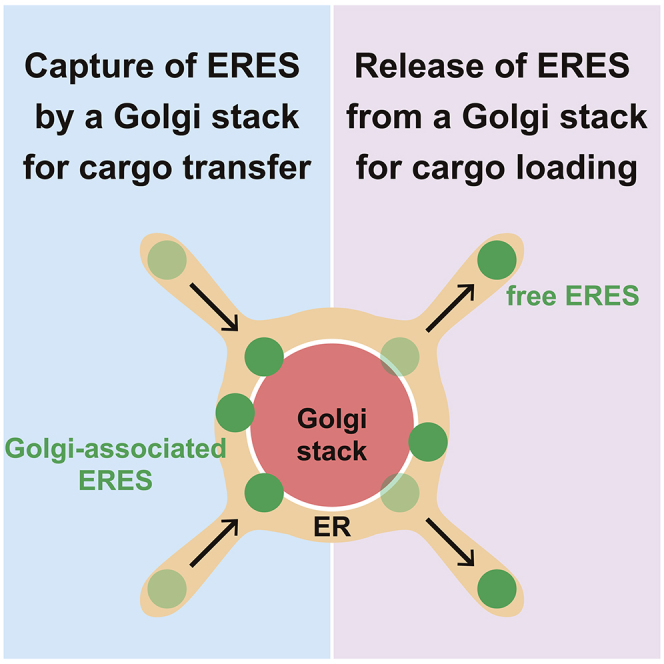

## Introduction

Protein transport from the endoplasmic reticulum (ER) to Golgi stacks is mediated by the coat protein complex II (COPII) machinery (Brandizzi and Barlowe, 2013). COPII vesicles are formed as follows: (1) The guanine nucleotide exchange factor SEC12 activates GTPase SAR1 (Barlowe and Schekman, 1993; Nakano and Muramatsu, 1989), (2) GTP-bound Sar1 recruits the inner-coat-protein complex SEC23/SEC24 to the ER membrane (Bi et al., 2002), (3) SEC24 functions in the selective loading of cargo proteins by recognizing their sorting signals (Miller et al., 2003), and then (4) SAR1-SEC23/SEC24-cargo complexes are polymerized by the outer-coat-protein complex SEC13/SEC31 to form COPII-coated vesicles (Bi et al., 2007; Stagg et al., 2008). COPII-coat-protein assembly occurs at ER subdomains called ER exit sites (ERESs) (Brandizzi and Barlowe, 2013), in which SEC16 functions as a scaffold and regulatory protein to facilitate the assembly (Connerly et al., 2005; Ivan et al., 2008; Kung et al., 2012; Watson et al., 2006; Yorimitsu and Sato, 2012). SEC16 and the plant homolog MAG5 are localized at ERESs but are not included in the COPII-coat complex (Takagi et al., 2013; Yorimitsu and Sato, 2012).

COPII vesicles bud off ERESs and then fuse with Golgi stacks to transfer cargo proteins (Brandizzi and Barlowe, 2013). In *Saccharomyces cerevisiae*, in which ERESs are not always located adjacent to Golgi stacks, *cis*-Golgi cisternae transiently come in contact with ERESs for COPII-dependent cargo transfer (Kurokawa et al., 2014). This result emphasizes the need for Golgi-stack contact with ERESs for the accurate transport of cargo proteins. On the other hand, unlike *S*. *cerevisiae*, ERESs are closely associated with a Golgi stack in various organisms: plants (daSilva et al., 2004; Ito et al., 2012; Takagi et al., 2013), *Drosophila melanogaster* (Ivan et al., 2008; Kondylis and Rabouille, 2003), *Caenorhabditis elegans* (Witte et al., 2011), *Pichia pastoris* (Bevis et al., 2002), and *Trypanosoma brucei* (Sevova and Bangs, 2009). In mammalian cells, ER-Golgi intermediate compartments (ERGICs) are closely localized to ERESs, instead of Golgi stacks forming a complex Golgi ribbon that is distant from ERESs (Hammond and Glick, 2000; Hughes et al., 2009). However, even though many studies have examined ER-Golgi cargo transport, the dynamic relationship between ERESs and Golgi stacks remains unknown.

A significant feature of plant cells is that cytosolic organelles including the ER are confined to a thin region between the plasma membrane and the membrane of a large central vacuole that occupies most of the cell volume. ERESs are distributed in this narrow region, which makes it possible to track their dynamic movements over a broad focal plane of the cell. To track ERESs in real time, we used variable-angle epifluorescence microscopy (VAEM), which provides high temporal resolution of events near the plasma membrane of a tissue (Konopka and Bednarek, 2008).

In this study, using VAEM and plant cells, we were able to observe the dynamic capture-and-release process of ERESs by Golgi stacks in *Arabidopsis thaliana*. Imaging of live cells revealed that some punctate ERESs gathered around a Golgi stack to form a beaded-ring structure at an ER network cavity, whereas other punctate ERESs were distributed on ER tubules and ER sheet rims. Real-time imaging with high temporal resolution showed that some punctate ERESs were captured and released by Golgi stacks in a cytoskeleton-independent manner. The present results reveal the dynamic cycling of ERESs responsible for the efficient cargo transfer from the ER to Golgi stacks.

## Results

### VAEM Images Reveal Minimal Punctate ERESs, Some of Which Appear to Form Beaded Rings

**Figure 1:**
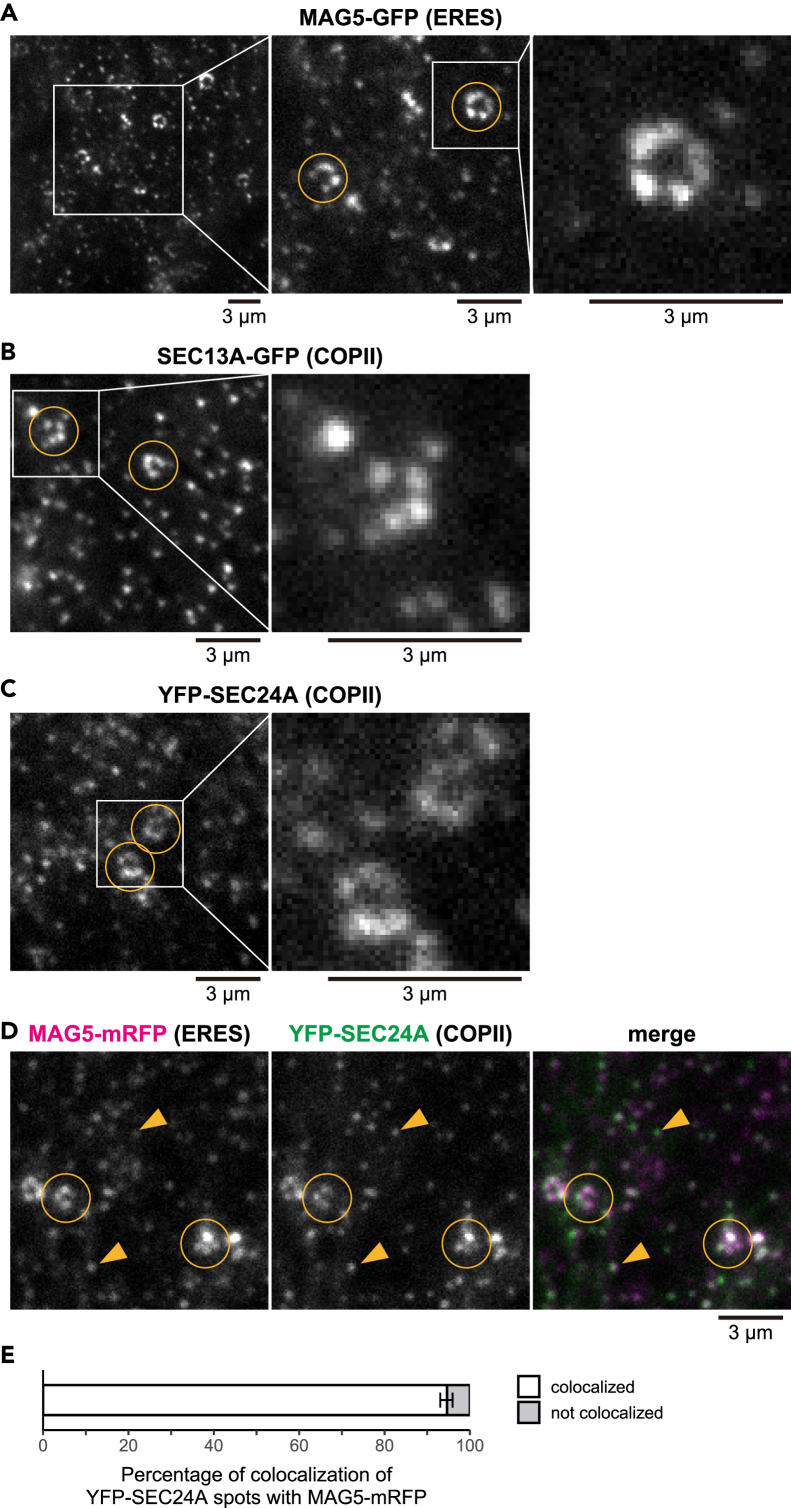
VAEM Images Showing Minimal Punctate ER Exit Sites (ERESs) that Are Accompanied with COPII Components This figure shows VAEM images of the surface of cotyledon epidermal cells of transgenic plants. Circles indicate beaded rings of punctate ERESs, and arrowheads indicate individually distributed punctate ERESs. Scale bars, 3 μm. (A) Images of ProMAG5:MAG5-GFP (ERES marker) in *mag5-1* plants. Center panel shows enlarged image of square in left panel; right panel shows enlarged image of square in middle panel. (B) Images of SEC13A-GFP (COPII marker). Right panel shows enlarged image of square in left panel. (C) Images of YFP-SEC24A (COPII marker). Right panel shows enlarged image of square in left panel. (D) Representative images of MAG5-mRFP (ERES marker) and YFP-SEC24A (COPII marker). See also Figure S1 for additional two biological replicates with similar results. (E) Proportions of YFP-SEC24A (COPII marker)-positive punctate structures that were colocalized and not colocalized with MAG5-mRFP (ERES marker). Error bars represent 95% confidence intervals. *n* = 1,011 YFP-SEC24A-labeled punctate structures from four biological replicates.

To identify ERESs, we used the ERES marker MAG5 labeled with GFP in a *MAG5*-deficient mutant under control of the *MAG5* promoter (Takagi et al., 2013). MAG5/SEC16A is a plant homolog of SEC16 that is not present in COPII vesicles (Takagi et al., 2013; Yorimitsu and Sato, 2012). VAEM analysis of the epidermal cell surfaces of cotyledons of the transgenic plants showed a number of MAG5-GFP-positive punctate ERESs with apparent sizes of 300–500 nm (Figure 1A). Most of the MAG5-GFP-positive punctate ERESs were individually distributed throughout the cells (Figure 1A, left). On the other hand, some of the punctate ERESs appeared to form beaded rings with diameters of ∼1.5 μm (Figure 1A, middle and right).

Next, we focused on COPII-coat proteins that are assembled at ERESs. Two COPII markers, SEC13A-GFP (Takagi et al., 2013) and YFP-SEC24A (Nakano et al., 2009), were selected because (1) overexpression of SEC24 has no harmful effects on protein transport from the ER (Hanton et al., 2007) and (2) overexpression of SEC24 or SEC13 does not induce ERES formation (Hanton et al., 2009). We investigated transgenic plants that stably expressed each of the COPII markers. VAEM analysis of the epidermal cell surface of cotyledons revealed that each COPII marker labeled punctate structures similar to the MAG5-GFP-positive punctate ERESs (Figures 1B and 1C). To determine the relationship between the COPII-positive punctate structures and punctate ERESs, transgenic plants that stably expressed both YFP-SEC24A and MAG5-mRFP were inspected by VAEM (Figures 1D and S1). Quantitative analysis showed that almost all (∼95%) of the YFP-SEC24A-positive punctate structures were labeled with MAG5-mRFP, indicating that COPII-coat proteins assemble on the punctate ERESs (Figure 1E). Hence, the MAG5-positive punctate ERESs can be regarded as a minimal ERES unit at which COPII components are assembled.

### Punctate ERESs Occur as Both Golgi-Associated and Golgi-Free ERESs

**Figure 2:**
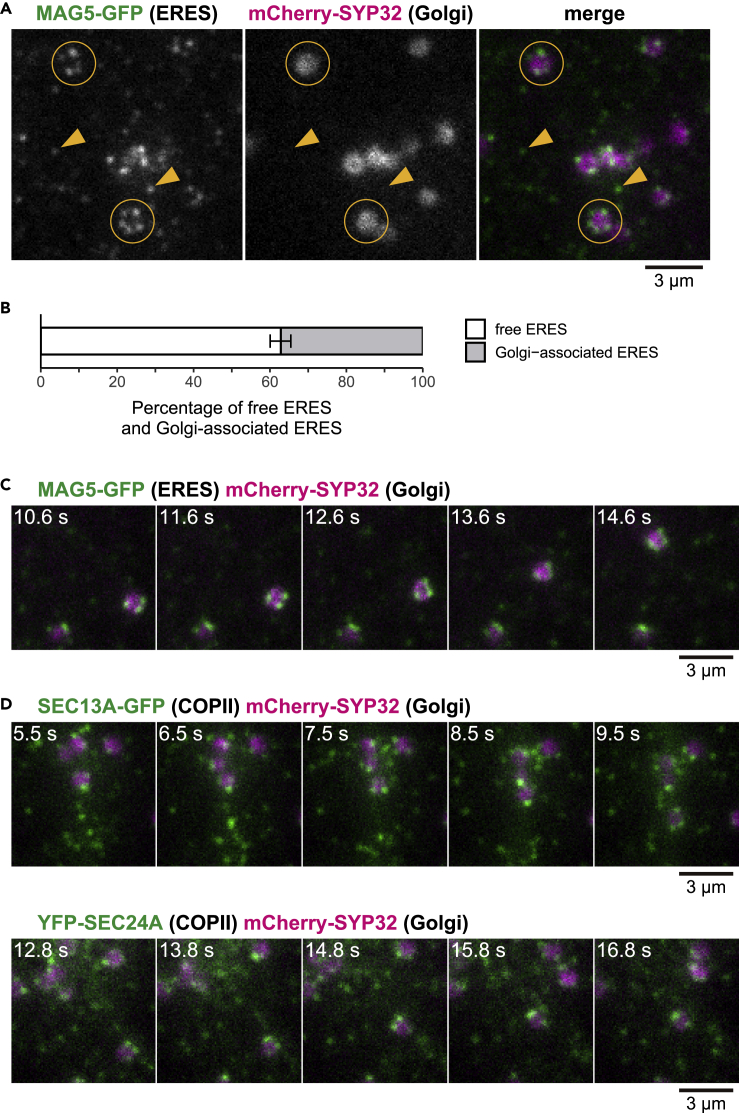
Punctate ERESs Occur as Golgi-Associated and Golgi-Free ERESs This figure shows VAEM images of the surface of cotyledon epidermal cells of transgenic plants. Scale bars, 3 μm. (A) Representative images of MAG5-GFP (ERES marker) and mCherry-SYP32 (Golgi marker). Circles indicate beaded rings of ERESs surrounding Golgi stack (Golgi-associated ERESs), and arrowheads indicate individually distributed ERESs free from Golgi stacks (Golgi-free ERESs). (B) Proportions of Golgi-associated ERESs and Golgi-free ERESs to total MAG5-GFP-labeled punctate ERESs. Error bars represent 95% confidence intervals. *n* = 1,066 MAG5-GFP-labeled punctate ERESs from five biological replicates. See also Figure S2 for image processing procedures for extraction of Golgi-free ERESs and Golgi-associated ERESs. (C) Time series of images of MAG5-GFP (ERES marker) and mCherry-SYP32 (Golgi marker) for 4.0 s. See also Video S1 for the original real-time movie. (D) Time series of images of mCherry-SYP32 (Golgi marker) and each of COPII markers, SEC13A-GFP (upper panels) and YFP-SEC24A (lower panels), for 4.0 s.

Positioning ERESs close to Golgi stacks might help to achieve efficient protein transport from the ER to *cis-*Golgi cisternae (Kurokawa et al., 2014). The relationship between the punctate ERESs and *cis-*Golgi cisternae was examined in transgenic plants that stably expressed the ERES marker MAG5-GFP and the *cis*-Golgi marker mCherry-SYP32 (Geldner et al., 2009). SYP32 is a *cis*-Golgi-localized SNARE (soluble *N*-ethylmaleimide sensitive factor attachment protein receptor) protein responsible for vesicle fusion with the target membrane (Uemura et al., 2004). VAEM showed that the beaded ring ERESs structures consisted of a ring of punctate ERESs that surrounded *cis*-Golgi cisternae (Figure 2A, circled). The beaded ring structures correspond to previously identified annular structures of Golgi-associated ERESs that were detected by confocal laser scanning microscopy (Ito et al., 2012; Takagi et al., 2013). On the other hand, most of the free punctate ERESs were distant from *cis*-Golgi cisternae (Figure 2A, arrows). Thus, the cells have two populations of punctate ERESs: a Golgi-associated population and a Golgi-free population. A quantitative analysis of punctate ERES images (Figure S2; see Transparent Methods for details) showed that the number of Golgi-free ERESs is around 1.7 times greater than the number of Golgi-associated ERESs (Figure 2B).

Real-time imaging of the transgenic plants showed that the Golgi stacks rapidly moved within the cell (Figure 2C; Video S1) with velocities similar to those of actin-dependent cytoplasmic streaming (see Figure 5B) (Boevink et al., 1998; Nebenfuhr et al., 1999). Even during their movement, punctate ERESs continued to be associated with Golgi stacks (Figure 2C; Video S1). Similar results were obtained with transgenic plants that stably expressed a COPII marker (SEC13A-GFP or YFP-SEC24A) and the *cis*-Golgi marker mCherry-SYP32 (Figure 2D). Each Golgi stack was accompanied by COPII-positive punctate structures, even while they moved. These results suggest that COPII component-bound punctate ERESs are interacting in some physical way with Golgi stacks.

Video S1. Golgi Stacks Remain Associated with the Surrounding ERESs during their Movement, Related to Figure 2C Real-time movies of the surface of cotyledon epidermal cells of seedlings expressing MAG5-GFP (ERES marker) and mCherry-SYP32 (Golgi marker). Time-sequential images were taken by VAEM at 100-ms intervals for 20 s. Scale bar, 2 μm.

### Free Punctate ERESs Are Preferentially Localized to ER Tubules and Sheet Rims

**Figure 3:**
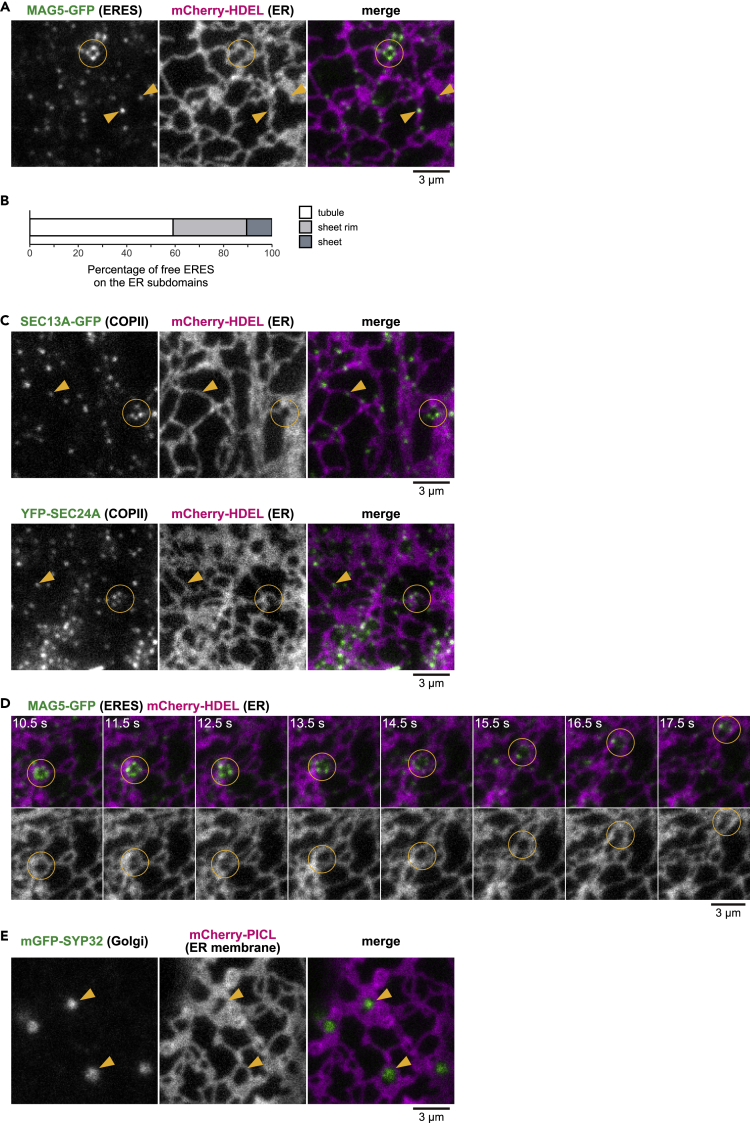
Golgi-Free ERESs Are Preferentially Localized to ER Tubules and Sheet Rims, whereas Golgi-Associated ERESs Are Localized on ER Membranes that Form ER-Network Cavities This figure shows VAEM images of the surface of cotyledon epidermal cells of transgenic plants. Circles indicate beaded rings of ERESs (Golgi-associated ERESs). Scale bars, 3 μm. (A) Representative images of MAG5-GFP (ERES marker) and mCherry-HDEL (ER marker) in the same field. Arrowheads indicate individually distributed ERESs (Golgi-free ERESs). (B) Proportional distribution of Golgi-free ERESs on each of ER subdomains (ER tubule, ER-sheet rim, and ER sheet) to total Golgi-free ERESs. *n* = 856 Golgi-free ERESs from five biological replicates. See also Figure S3 for image processing procedures for extraction of the ER subdomains and Golgi-free ERESs. (C) Images of mCherry-HDEL (ER marker) and each of COPII markers, SEC13A-GFP (upper panels) and YFP-SEC24A (lower panels). Arrowheads indicate individually distributed ERESs (Golgi-free ERESs). (D) Time series of images of MAG5-GFP (ERES marker) and mCherry-HDEL (ER marker) for 7.0 s. Merged images (upper) and single-labeled images of mCherry-HDEL (lower) are shown. See also Video S2 for the original real-time movie. (E) Images of mGFP-SYP32 (Golgi marker) and mCherry-PICL (ER membrane marker). Note that Golgi stacks are localized to the ER cavities (arrowheads).

The topological relationship of punctate ERESs with the ER was investigated in transgenic plants that stably expressed the ERES marker MAG5-GFP and the ER marker mCherry-HDEL (Nelson et al., 2007). Free punctate ERESs were widely distributed throughout the ER network (Figure 3A, arrows). Similar results were obtained with COPII-markers (SEC13A-GFP and YFP-SEC24A) (Figure 3C, circled). The ER network is composed of tubules and sheets (Friedman and Voeltz, 2011). To determine the distribution of free punctate ERESs, three typical ER structures (tubules, sheets, and sheet rims; see Figure 6A) were extracted from original fluorescence images of the ER by semi-automatic image processing techniques (Haralick et al., 1987; Kimori, 2011) (Figure S3 and Transparent Methods for details). Approximately 90% of detected free punctate ERESs were on the ER tubules and sheet rims (Figure 3B). Free punctate ERESs are preferentially localized on the curved structures of ER membrane. The free punctate ERESs were associated with COPII markers (Figure 1D), which indicates that COPII vesicles emerge from free punctate ERESs on the ER tubules and sheet rims of the ER network. This is consistent with a previous finding in *Nicotiana benthamiana* that SEC12, which is responsible for initiating COPII vesicle formation, is distributed throughout the ER membrane (daSilva et al., 2004).

### Golgi-Associated Punctate ERESs Are Localized on ER Membranes that Form ER Network Cavities

In contrast to preferential localization of free punctate ERESs on the ER tubules and sheet rims, beaded ring-shaped structures labeled with the ERES marker were detected in ER network cavities (Figure 3A, circled). This result indicates that ring-shaped COPII component-bound punctate ERESs, which correspond to Golgi-associated punctate ERESs, are exclusively localized to the ER network cavities. The localization of Golgi-associated punctate ERESs is consistent with previous studies: ERESs have been localized on cup-like structures of the ER in plants (Takagi et al., 2013), on cup-shaped domains of the ER in mammalian cells (Hughes et al., 2009), and on saddle-like structures of the ER in *S*. *cerevisiae* (Okamoto et al., 2012). Golgi-associated punctate ERESs that were confined to the ER cavities were stable and did not disassemble while they moved in a cytoplasmic streaming-dependent manner (Figure 3D, circled; Video S2). These results suggest that, on the ER membrane forming the cavities, COPII component-bound punctate ERESs are stably associated with Golgi stacks.

Video S2. Golgi-Associated ERESs Remain Localized at the ER Cavity during Their Movement, Related to Figure 3D Real-time movies of the surface of cotyledon epidermal cells of seedlings expressing MAG5-GFP (ERES marker) and mCherry-HDEL (ER marker). Time-sequential images were taken by VAEM at 100-ms intervals for 20 s. Scale bar, 2 μm.

To more precisely localize ERESs on the ER membrane, we focused on PICL-TMD, a transmembrane domain of the ER membrane protein PAMP-INDUCED COILED-COIL LIKE (Venkatakrishnan et al., 2013), instead of the ER luminal marker mCherry-HDEL and generated transgenic plants that stably coexpressed the ER membrane marker mCherry-PICL-TMD and the *cis*-Golgi marker mGFP-SYP32. In the cells, Golgi stacks were localized to the ER cavities (Figure 3E). These results indicate that some punctate ERESs are localized on the parts of the ER membranes that form ER cavities.

### Cycling of Punctate ERESs between Golgi-Bound and Golgi-Free States

**Figure 4:**
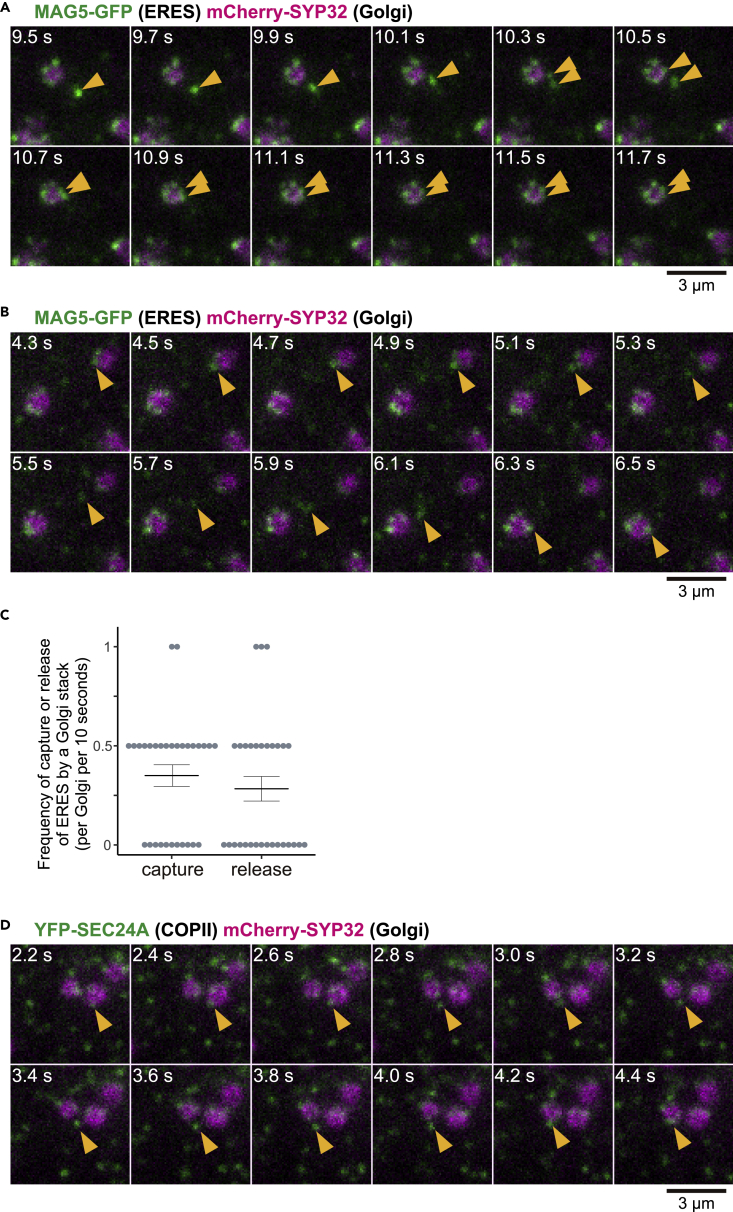
Cycling of Punctate ERESs between Golgi-Bound and Free States This figure shows VAEM images of the surface of cotyledon epidermal cells of transgenic plants. Scale bars, 3 μm. (A) Time series of representative images of MAG5-GFP (ERES marker) and mCherry-SYP32 (Golgi marker) for 2.2 s. Arrowheads indicate punctate ERESs being captured by a Golgi stack. See also Video S3 (left) for the original real-time movie. (B) Time series of representative images of MAG5-GFP (ERES marker) and mCherry-SYP32 (Golgi marker) for 2.2 s. Arrowheads indicate a punctate ERES being released by a Golgi stack and then captured by another Golgi stack. See also Video S3 (right) for the original real-time movie. (C) Frequencies of the events of capture and release of punctate ERESs for 10 s by a single Golgi stack. Data are represented as mean ± SEM (*n* = 30 Golgi stacks from 10 biological replicates). (D) Time series of representative images of YFP-SEC24A (COPII marker) and mCherry-SYP32 (Golgi marker) for 2.2 s. Arrowheads indicate a punctate ERES being released by a Golgi stack and then captured by another Golgi stack. See also Video S4 for the original real-time movie.

The dynamics of punctate ERESs was examined with high temporal resolution of cells that coexpressed the ERES marker MAG5-GFP and the *cis*-Golgi marker mCherry-SYP32. Some free punctate ERESs were captured by a Golgi stack to directly become punctate ERESs in a Golgi-associated state (Figure 4A; Video S3, left). In another image, a punctate ERES in a Golgi-bound state was released to become a punctate ERES in a free state, which was then captured by another Golgi stack (Figure 4B; Video S3, right). ERES captures and ERES releases by a Golgi stack were each observed about once in 30–40 s (Figure 4C), although the frequencies might be underestimated because of the difficulty of tracking all events by VAEM. Similarly, in the transgenic plants expressing the COPII marker YFP-SEC24A and the *cis*-Golgi marker, a COPII component-bound punctate ERES released by a Golgi stack was captured by another Golgi stack (Figure 4D; Video S4). These results indicate that punctate ERESs cycle between bound and free states. Thus, Golgi stacks might have an ability to dynamically capture and release punctate ERESs.

Video S3. Capture and Release of a Punctate ERES by a Golgi Stack, Related to Figure 4A and 4B Real-time movies of the surface of cotyledon epidermal cells of seedlings expressing MAG5-GFP (ERES marker) and mCherry-SYP32 (Golgi marker). Time-sequential images were taken by VAEM at 100-ms intervals for 20 s. Scale bar, 2 μm.

Video S4. Capture and Release of a COPII-Component-Bound ERES by a Golgi Stack, Related to Figure 4D Real-time movies of the surface of cotyledon epidermal cells of seedlings expressing YFP-SEC24A (COPII marker) and mCherry-SYP32 (Golgi marker). Time-sequential images were taken by VAEM at 100-ms intervals for 20 s. Scale bar, 2 μm.

**Figure 5:**
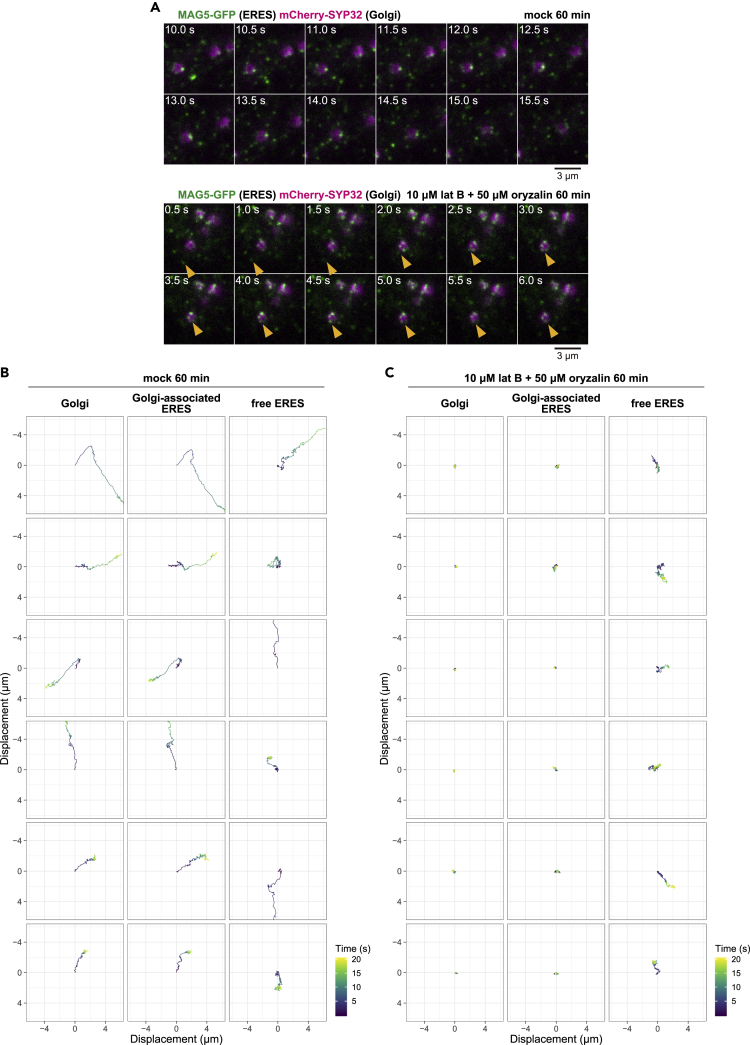
Punctate-ERES Cycling Is Independent of a Cytoskeleton-Dependent Movement (A) Time series of representative VAEM images of MAG5-GFP (ERES marker) and mCherry-SYP32 (Golgi marker) for 5.5 s. The cotyledon cells were mock-treated (upper panels) or treated with cytoskeleton inhibitors (10 μM latrunculin B and 50 μM oryzalin) (lower panels) for 60 min. See also Video S5 (left and right) for the original real-time movies of mock- and inhibitor-treated cells, respectively. Scale bars, 3 μm. (B and C) Six example trajectories of fluorescence particles corresponding to each of Golgi stack, Golgi-associated ERES, and free ERES in cotyledon cells that expressed MAG5-GFP (ERES marker) and mCherry-SYP32 (Golgi marker). The cotyledon cells were mock-treated (B) or treated with the cytoskeleton inhibitors (C) for 60 min. Time-coded trajectories of the fluorescence particles were tracked from the center of each two-dimensional lattice. Three biological replicates were performed.

Both Golgi stacks (Boevink et al., 1998; Nebenfuhr et al., 1999) and ER strands (Ueda et al., 2010) have been observed to move along cytoplasmic streaming in a cytoskeleton-dependent manner. On the other hand, ER-to-Golgi protein transport does not depend on actin or microtubules (the components of the cytoskeleton) (Brandizzi et al., 2002). To examine the involvement of the cytoskeleton in the cycling of punctate ERESs, transgenic plants were treated with a mixture of an actin-depolymerizing reagent (latrunculin B) and a microtubule-depolymerizing reagent (oryzalin). In the control cells treated with the solvent dimethyl sulfoxide, punctate ERESs showed directional movements along cytoplasmic streaming and random movements similar to Brownian motion (Figure 5A, upper; Video S5, left). Fluorescence particles corresponding to individual Golgi stacks, Golgi-associated ERESs, and free ERESs were tracked on the X-Y plane (Figure 5B). When the inhibitors were used, the directional movements of Golgi-associated and free punctate ERESs were completely impaired (Figure 5A, lower; Video S5, right). However, in the same cells treated with the inhibitors, free punctate ERESs randomly moved and reached Golgi stacks to become Golgi-associated punctate ERESs (Figure 5A, lower, arrowheads). Time-coded trajectories of fluorescence particles of free ERESs clearly show they moved randomly (Figure 5C). Hence, Golgi stacks are able to capture punctate ERESs independently of the cytoskeleton (actin filaments and microtubules).

Video S5. Cycling of Punctate ERESs Occurs Independently of the Cytoskeleton-Dependent Movement, Related to Figure 5 Real-time movies of the surface of cotyledon epidermal cells of seedlings expressing MAG5-GFP (ERES marker) and mCherry-SYP32 (Golgi marker) treated with mock (left) or with 10 μM latrunculin B and 50 μM oryzalin (right) for 60 min. Time-sequential images were taken by VAEM at 100-ms intervals for 20 s. Scale bar, 2 μm.

## Discussion

Based on the findings with COPII markers, two ER-Golgi transport models have been proposed in plants (Brandizzi, 2018; Ito et al., 2014). In the kiss-and-run model (also called the stop-and-go model or the dock, pluck, and go model) (Nebenfuhr et al., 1999; Staehelin and Kang, 2008; Yang et al., 2005), ERESs are transiently associated with Golgi stacks whose movements have temporarily stopped. However, our observations show that the Golgi-associated punctate ERESs are moving (Figures 2C and 2D). In the secretory unit model, the secretory units are stable architecture composed of ERESs and Golgi stacks, which predominantly functions in the transport to mobile Golgi stacks (daSilva et al., 2004). In this model, Golgi-free ERESs (if any) can be regarded as premature secretory units (Brandizzi, 2018; Robinson et al., 2015). Our observations have a difference from the secretory unit model: dynamic cycling of punctate ERESs between the Golgi-bound and Golgi-free states (Figure 4). A hybrid model that incorporates different features of the kiss-and-run and secretory unit models has been also proposed (Ito et al., 2014) but never demonstrated.

**Figure 6:**
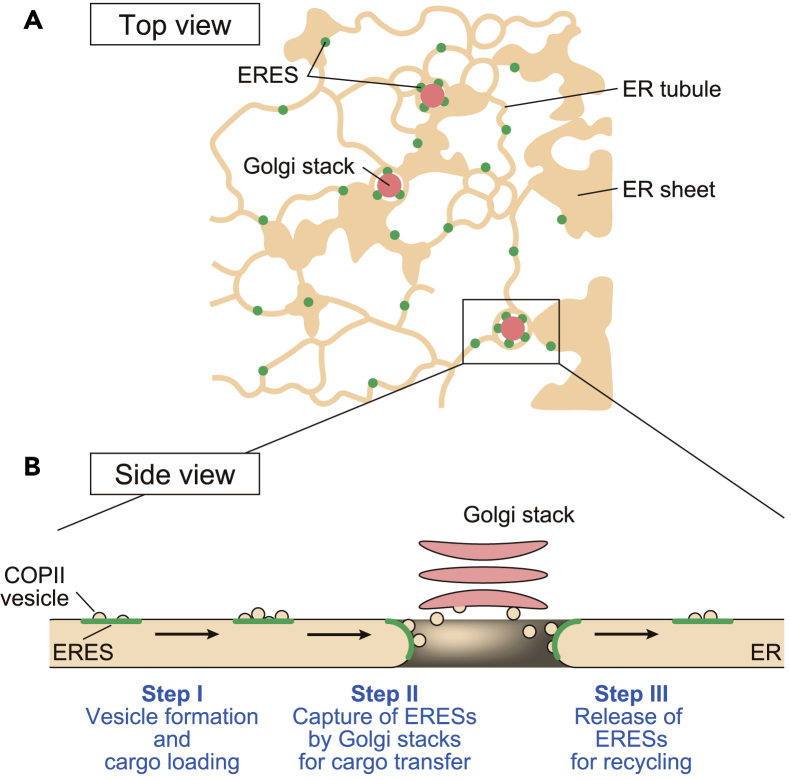
A Hypothetical Model of Capture-and-Release of Punctate ERESs by Golgi Stacks (A) Top view illustrating the distribution of punctate ERESs (green dots) on the ER network (orange) and Golgi stacks (magenta closed circles). Punctate ERESs exist in both Golgi-free and Golgi-associated states. Most of Golgi-free ERESs are individually distributed on the ER tubules and ER sheet rims, whereas Golgi-associated ERESs are distributed in the ER cavities. (B) Side view of the ER-Golgi interface illustrates a model for the dynamic capture and release of ERESs by Golgi stacks, in which the ER-Golgi transport occurs in three steps. In Step I, ERESs with forming COPII vesicles (green dots) move around the ER network (orange) to load *de novo* synthesized cargo proteins. In Step II, COPII-bound ERESs are captured by a Golgi stack (pink) in an ER cavity, where COPII vesicles bud from ERESs and fuse with *cis*-Golgi cisternae to transport proteins. In Step III, ERESs are released by Golgi stacks for recycling.

Based on the present findings with an authentic ERES marker (MAG5), we propose a model for the dynamic capture-and-release of ERESs by Golgi stacks (Figure 6) that overcomes these problems. In this model, the capture and release occurs in three steps: (1) punctate ERESs associated with COPII components move around the ER membrane to load *de novo* synthesized cargo proteins, (2) COPII-bound punctate ERESs that wander into an ER cavity are captured by a Golgi stack, and (3) punctate ERESs are released by Golgi stacks for recycling. In *S*. *cerevisiae*, COPII vesicles bud off from ERESs during transient contact with Golgi cisternae (Kurokawa et al., 2014). Electron microscopy of *A*. *thaliana* cells showed that more than 95% of COPII vesicles were detected near Golgi stacks (Kang and Staehelin, 2008). These results suggest that COPII vesicles bud and fuse with *cis*-Golgi cisternae to transport cargo proteins in step 2 of our hypothetical model (Figure 6).

In tobacco BY-2 cells treated with brefeldin A, the *cis*-most cisternae of Golgi are not redistributed into the ER and are associated with ERESs (Ito et al., 2012), suggesting that the *cis*-most cisternae of Golgi are better designed for capturing ERESs in plants. At the ER-Golgi interface, TFG complexes link ERESs to ERGIC membranes in mammalian cells (Johnson et al., 2015) and Tango1 contributes to the organization of the ERES-Golgi interface in mammalian cells (Santos et al., 2015) and *D*. *melanogaster* (Liu et al., 2017). Although *A*. *thaliana* has no orthologs to TFG and Tango1, their functional homologs might be involved in the process of capture and release of ERESs by Golgi stacks. On the other hand, ERES structures similar to beaded ring-shaped ERESs were also detected in mammalian cells (McCaughey et al., 2016) and in *D*. *melanogaster* (Liu et al., 2017). Additionally, a simulation of the dynamics of ERESs shows that ERESs randomly move around the ER tubules and are eventually confined by cup-shaped domains of the ER in mammalian cells (Stadler et al., 2018), as in *A*. *thaliana* (Figure 6). Thus, our model of capture and release of punctate ERESs by Golgi stacks might be applicable to the ER-to-Golgi transport in mammals and in *Drosophila*.

### Limitations of the Study

Our study has four limitations. We were unable to (1) verify the dynamics of *individual* COPII vesicles during the budding off of ERESs and fusing with Golgi stack membrane because the close proximity of ERESs and Golgi stacks made it difficult to detect individual COPII vesicles located between them; (2) track cargo-protein transport from the ER to Golgi stacks due to a lack of appropriate pulse-chase type of experimental systems in plant cells; (3) determine the exact frequency of the capture-and-release events of ERESs by a Golgi stack because the ERESs move away from the focal plane and overlap each other during their movement; (4) quantify the changes in signal intensity of COPII on ERESs, due to the ERES movements and a wavelength-dependent control system of laser angles of VAEM.

### Resource Availability

#### Lead Contact

Further information and requests for resources and reagents should be directed to and will be fulfilled by the Lead Contact, Ikuko Hara-Nishimura (ihnishi@gr.bot.kyoto-u.ac.jp).

#### Materials Availability

Materials generated in this study are available from the Lead Contact with a completed Materials Transfer Agreement.

#### Data and Code Availability

The codes supporting the current study have not been deposited in a public repository because these are parts of further investigation but are available from the corresponding author on request.

## Methods

All methods can be found in the accompanying Transparent Methods supplemental file.

## Supplemental Information

Document S1. Transparent Methods and Figures S1–S3
