# Supplementary material for: Dynamic Capture and Release of Endoplasmic Reticulum Exit Sites by Golgi Stacks in Arabidopsis
Source: iScience. 2020 Jun 12;23(7):101265. doi: 10.1016/j.isci.2020.101265 (PMC7322076; doi:10.1016/j.isci.2020.101265)
Supplement: Document S1. Transparent Methods and Figures S1–S3 [file mmc1.pdf]

iScience, Volume *23*

Supplemental Information

Dynamic Capture and Release of Endoplasmic Reticulum Exit Sites by Golgi Stacks in Arabidopsis

Junpei Takagi, Yoshitaka Kimori, Tomoo Shimada, and Ikuko Hara-Nishimura

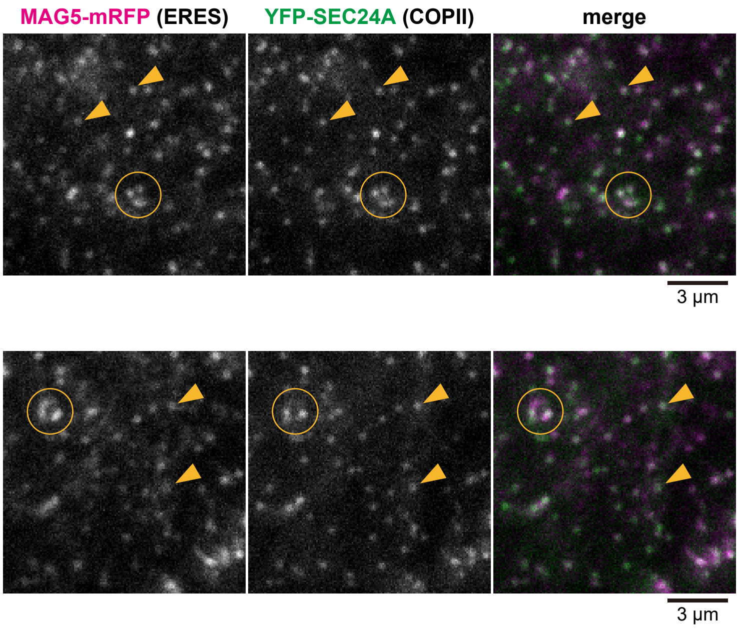

Figure S1. Almost all of YFP-SEC24A-Positive Punctate Structures Are Labeled with MAG5-mRFP, Related to Figure 1D.

Two sets of other representative VAEM images of MAG5-mRFP (ERES marker) and YFP-SEC24A (COPII marker). The surface of cotyledon epidermal cells of transgenic plants was examined. Circles indicate beaded rings of punctate ERESs (Golgi-associated ERESs) and arrowheads indicate individually-distributed punctate ERESs (Golgi-free ERESs). Bars = 3 μm.

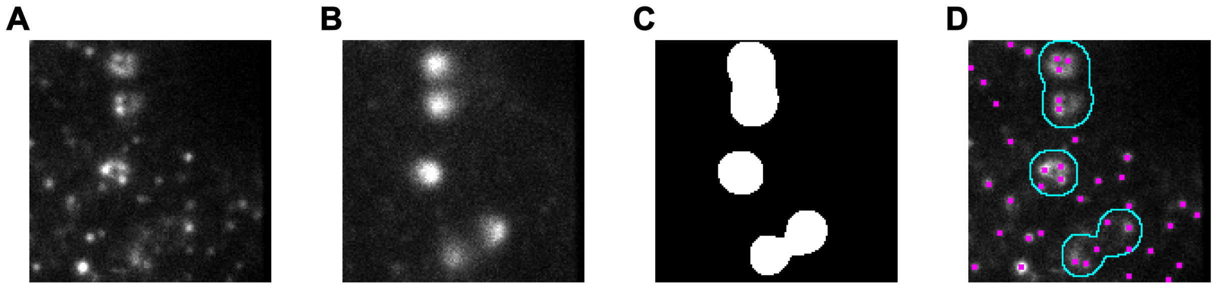

Figure S2. Image Processing Procedures for Extraction Free ERESs and Golgi-Associated ERESs, Related to Figure 2B.

(A) An original image of punctate ERESs.

(B) An original image of Golgi stacks.

(C) Binarized image of (B). The Golgi stack regions are shown in white.

(D) Final image after applying image processing steps. The peaks of the fluorescent spots of ERESs are shown in magenta, and the outlines of the Golgi stack regions are shown in cyan. The ERES spots whose peaks were located in the Golgi stack regions are defined as Golgi-associated ERESs, while the others are free ERESs.

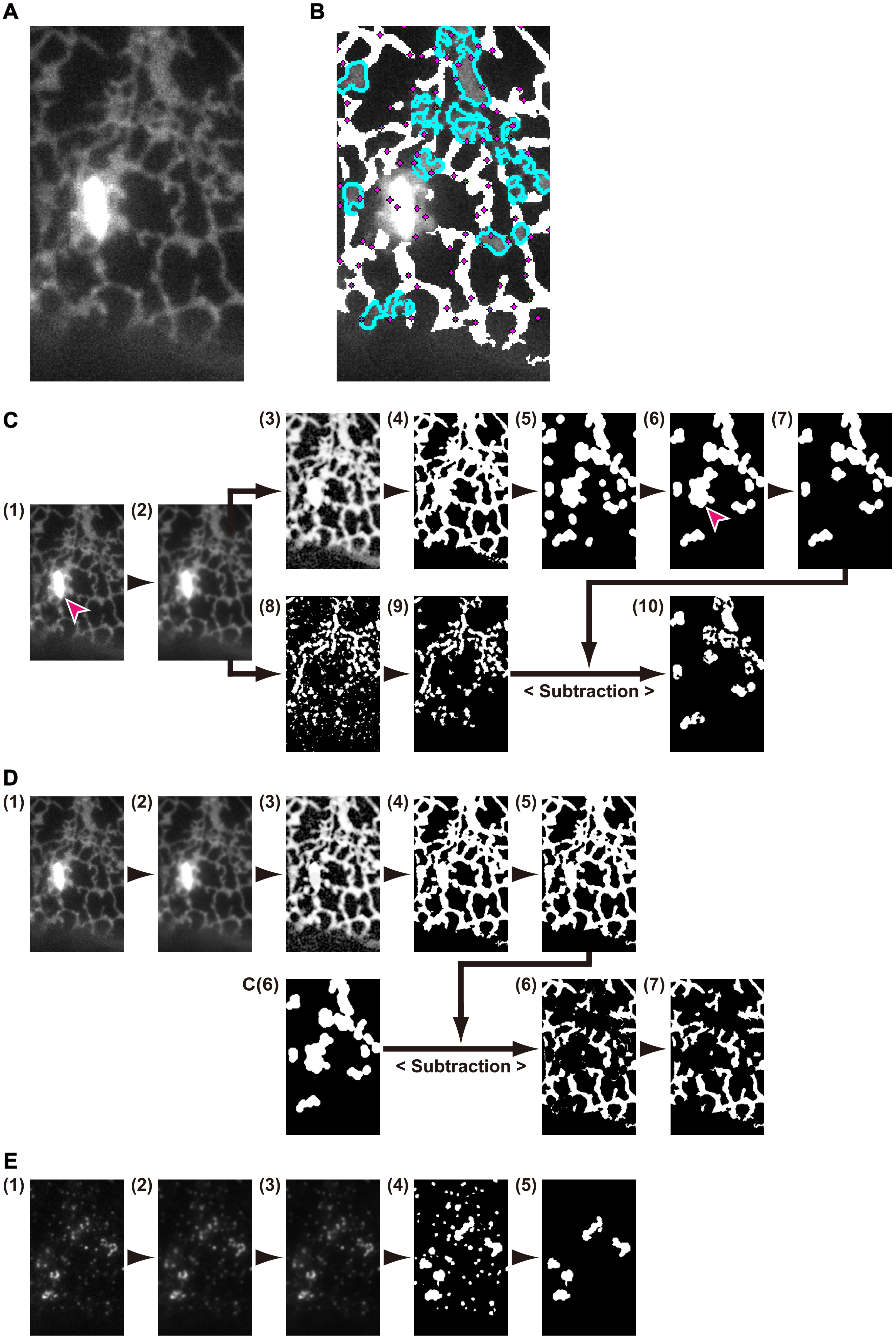
A
B
C
(1)
(2)
(3)
(4)
(5)
(6)
(7)
(8)
(9)
(10)
< Subtraction >
D
(1)
(2)
(3)
(4)
(5)
C(6)
(6)
(7)
< Subtraction >
E
(1)
(2)
(3)
(4)
(5)

Figure S3. Image Processing Procedures for Extraction of the ER Structures and Punctate ERESs, Related to Figure 3B.

(A) An original image.

(B) Final image after applying our proposed image processing steps. The ER tubules are shown in white. The ER sheet rims are shown in cyan, which are defined as the contour of the ER sheet region with a width of three pixels. ERESs are represented in magenta. These structures are superimposed on the original image.

(C-E) Image processing steps for extraction of different ER regions and ERESs.

(C) ER sheet regions. (1) Example of the original image. This image is a partial region of the fluorescent microscopy image. The ER body region is indicated by an arrowhead. (2) The image resulting after Gaussian blurring to remove noise effects. (3) The image resulting after contrast enhancement based on white top-hat transform through rotational morphological processing (RMP). (4) The binarized image using an automatic thresholding method. (5) The image produced after the opening procedure with RMP to remove the ER tubule regions. (6) The resulting image after area opening operation to remove small isolated regions in the image (5). The ER body region, which is indicated by an arrowhead, required manual removal. (7) The image obtained after image processing. (8) The binarized image. Grooves seen in image (2), which are narrow regions with low intensities, were extracted by black top-hat transform through RMP and the resultant image is binarized. (9) The image resulting after area opening operation to remove small isolated regions. (10) The final ER sheet image after the completion of processing. Image (10) is obtained by subtracting image (9) from image (7).

(D) ER tubule regions. (1) An original image. (2) The image produced after Gaussian blurring. (3) The resulting image after white top-hat transform through RMP. (4) Image binarized using an automatic thresholding method. (5) The image resulted after area opening operation to remove small isolated regions. (6) The image resulted from subtracting image C (6) from image (5). (7) The final image showing the extracted ER tubule regions. The image is obtained by removing small, isolated regions present in the image (6).

(E) Free ERES and Golgi-associated ERES regions. (1) An original image. (2) Image produced after Gaussian blurring. (3) The image resulted after closing through RMP to connect adjacent ERESs smoothly. (4) The binarized image using an automatic thresholding method. (5) The image resulted after manual selection of Golgi-associated ERES regions. Fluorescence peaks were extracted from image (1) using the find maxima tool in ImageJ, and peaks other than those within the Golgi-associated ERES regions were defined as free ERESs.

Transparent Methods

Key Resource Table

REAGENT or RESOURCE	SOURCE	IDENTIFIER
**Bacterial and Virus Strains**		
A. tumefaciens GV3101	N/A	N/A
**Chemicals, Peptides, and Recombinant Proteins**		
Oryzalin PESTANAL®	Sigma-Aldrich	Cat#36182-100MG
Latrunculin B from *Latruncula magnifica*	Sigma-Aldrich	Cat#L5288-1MG
**Experimental Models: Organisms/Strains**		
Arabidopsis: ProMAG5:MAG5-GFP *mag5-1*	Hara-Nishimura Lab., (Takagi et al., 2013)	N/A
Arabidopsis: Pro35S:SEC13A-GFP	Hara-Nishimura Lab., (Takagi et al., 2013)	N/A
Arabidopsis: Pro35S:YFP-SEC24A	Hara-Nishimura Lab., (Nakano et al., 2009)	N/A
Arabidopsis: ProUBQ10:mCherry-SYP32	Geldner Lab., (Geldner et al., 2009)	WAVE22R
Arabidopsis: Pro35S:mCherry-HDEL	Nebenführ Lab., (Nelson et al., 2007)	ER-rb
Arabidopsis: ProMAG5:MAG5-GFP ProUBQ10:mCherry-SYP32 *mag5-1*	This paper	N/A
Arabidopsis: Pro35S:SEC13A-GFP ProUBQ10:mCherry-SYP32	This paper	N/A
Arabidopsis: Pro35S:YFP-SEC24A ProUBQ10:mCherry-SYP32	This paper	N/A
Arabidopsis: ProMAG5:MAG5-GFP Pro35S:mCherry-HDEL *mag5-1*	This paper	N/A
Arabidopsis: Pro35S:SEC13A-GFP Pro35S:mCherry-HDEL	This paper	N/A
Arabidopsis: Pro35S:YFP-SEC24A Pro35S:mCherry-HDEL	This paper	N/A
Arabidopsis: ProMAG5:MAG5-mRFP *mag5-1*	This paper	N/A
Arabidopsis: Pro35S:YFP-SEC24A ProMAG5:MAG5-mRFP *mag5-1*	This paper	N/A
Arabidopsis: ProSYP32:mGFP-SYP32 ProUBQ10:mCherry-PICL TMD	This paper	N/A

**Oligonucleotides**		
Primer : IF-SYP32-pF : AACCAATTCAGTCGACGCTGAAGAGAGAAATCGAAAACC	This paper	N/A
Primer : IF-SYP32-pR : GCCCTTGCTCACCATTATTCTTCCTCCAATTCCTTGGA	This paper	N/A
Primer : IF-SYP32-F : GGAGGCGGAGGTGCTATGTCGGCAAGGCATGGG	This paper	N/A
Primer : IF-SYP32-3UTRR : AAGCTGGGTCTAGATATCTTTCGTCTCTGTAGTGCCT	This paper	N/A
Primer : mGFP-F : ATGGTGAGCAAGGGCGAG	This paper	N/A
Primer : mGFP-L-R : AGCACCTCCGCCTCCACCCTTGTACAGCTCGTCCATGC	This paper	N/A
Primer : IF-proUBQ10-F : AACCAATTCAGTCGACAGTCTAGCTCAACAGAGCTTTTAAC	This paper	N/A
Primer : proUBQ10-R : GGCTGTTAATCAGAAAAACTCAGA	This paper	N/A
Primer : IF-PICL-TMD-F : GGAGGCGGAGGTGCTAAAGCTGAAACATGGCATCTCA	This paper	N/A
Primer : IF-PICL-TMD-R : AAGCTGGGTCTAGATATCAATAATTTTTCCCAACAATGA	This paper	N/A
Primer : IF-proUBQ10-mCherry-F : TTCTGATTAACAGCCATGTTGAGCAAGGGCGAGG	This paper	N/A
Primer : mCherry-L-R : AGCACCTCCGCCTCCACCCTTGTACAGCTCGTCCATGC	This paper	N/A
**Recombinant DNA**		
Pro35S:mCherry-HDEL	Nebenführ Lab., (Nelson et al., 2007)	ER-rb
ProMAG5:MAG5-mRFP	This paper	N/A
ProSYP32:mGFP-SYP32	This paper	N/A
ProUBQ10:mCherry-PICL TMD	This paper	N/A
**Software and Algorithms**		
Fiji (ImageJ)	NIH, (Schindelin et al., 2012)	https://fiji.sc/
MetaMorph (ver. 7.8.3.0)	Molecular Devices	https://www.moleculardevices.co.jp/

Plant Materials and Growth Conditions

Arabidopsis thaliana, accession Columbia-0 (Col-0), was used as the wild-type plant. The following transgenic lines used in this study were described previously: ProMAG5:MAG5-GFP *mag5-1* (Takagi et al., 2013), SEC13A-GFP (Takagi et al., 2013), and YFP-SEC24A (Nakano et al., 2009). mCherry-SYP32 [WAVE22R (Geldner et al., 2009)] and mCherry-HDEL [ER-rb (Nelson et al., 2007)] were provided by Dr. N. Geldner and Dr. A. Nebenführ, respectively. Seeds were surface-sterilized with 70% ethanol and then sown onto 1x MS medium that contained 0.5% [w/v] Gellan Gum (Wako), 1% [w/v] sucrose, 1x Murashige-and-Skoog medium (Wako), and 2.5 mM MES-KOH, pH 5.7. The seeds were incubated at 4 °C for 2 d to break dormancy, followed by growth at 22 °C under continuous light.

Live Cell Imaging

Fluorescence images from cotyledon epidermal cells of 3-d-old seedlings were captured using an inverted fluorescence microscope (IX83; Olympus) equipped with a total internal reflection fluorescence microscopy system (IX2-RFAEVA-2; Olympus), solid-state lasers at 488 nm (Sapphire 488 LP; Coherent) and 561 nm (Sapphire 561 LP; Coherent), 100x oil immersion objectives (UApoN 100x OTIRF, numerical aperture (NA) = 1.49; UPlanApo 100x OHR, NA = 1.50; Olympus), Dual View 2 emission splitting system (Photometrics) and a EM-CCD camera (iXon3 897; Andor). For real-time imaging, time-sequential images were captured at 100 ms intervals for 10-20 s. Images were processed using MetaMorph (Molecular Devices) and Fiji (Schindelin et al., 2012).

Molecular Cloning

Genomic DNA of wild-type plants was used to PCR amplify the following regions: 2.0 kb of the *SYP32* upstream sequence, 2.3 kb sequence including the coding region and 3' UTR of *SYP32*, 2.0 kb of the *UBQ10* promoter, and the C-terminal region of *PICL* (see Key Resource Table). mGFP (Segami et al., 2018) and mCherry (Shaner et al., 2004) were also amplified by PCR (see Key Resource Table). The amplified fragments were inserted into pENTER 1A dual selection (Invitrogen) using an In-Fusion HD cloning kit (Clontech) to produce the ProSYP32:mGFP-SYP32 and ProUBQ10:mCherry-PICL TMD. ProSYP32:mGFP-SYP32 and ProUBQ10:mCherry-PICL TMD were transferred into the destination vectors pBGW (Karimi et al., 2002) and pGWB601 (Nakamura et al., 2010) using LR clonase II

(Invitrogen), respectively. For the expression of MAG5-mRFP driven by the native promoter, the LR recombination reaction was performed to transfer the fragment of ProMAG5:MAG5Δstop (Takagi et al., 2013) into the destination vector pGWB653 (Nakamura et al., 2010). pFGC19/mCherry-HDEL was described previously (Nelson et al., 2007). These binary vectors were introduced into *Agrobacterium tumefaciens* (strain GV3101) by electroporation. The binary vectors were transformed into wild-type, *mag5-1* or ProMAG5:MAG5-GFP *mag5-1* plants with *Agrobacterium* using the floral dip method (Clough and Bent, 1998) to generate ProSYP32:mGFP-SYP32 ProUBQ10:mCherry-PICL-TMD, ProMAG5:MAG5-mRFP *mag5-1* or ProMAG5:MAG5-GFP Pro35S:mCherry-HDEL *mag5-1*. T1 seeds were selected in 1x MS medium containing 10 mg/L of BASTA to establish independent transgenic lines. The following transgenic lines used in this study were generated by crossing: ProMAG5:MAG5-GFP mCherry-SYP32 *mag5-1*, SEC13A-GFP mCherry-SYP32, YFP-SEC24A mCherry-SYP32, SEC13A-GFP mCherry-HDEL, YFP-SEC24A mCherry-HDEL, and YFP-SEC24A ProMAG5:MAG5-mRFP *mag5-1*.

Pharmacological Treatment

Latrunculin B and oryzalin stock solutions (5 mM and 50 mM, respectively) were made in DMSO. Three-day-old seedlings were incubated in water containing 0.3% DMSO (mock) or 10 µM latrunculin B and 50 µM oryzalin (Lat B and Oryzalin) for 1 h. The seedlings were observed immediately after the treatment.

Quantitative Analysis of Colocalization of COPII and ERES Markers

Fluorescence peaks of spots labeled with YFP-SEC24A (COPII marker) and MAG5-mRFP (ERES marker) were detected using the find maxima tool in ImageJ. To minimize detection errors, each of fluorescence spots of YFP-SEC24A was manually verified by referring to its time-sequential image. The YFP-SEC24A spot whose peak was positioned in a square region of 5 x 5 pixels (400 × 400 nm) centered at MAG5-mRFP peak was defined as colocalized.

Quantitative Analysis of Free ERESs and Golgi-Associated ERESs

Fluorescence peaks of ERES (MAG5-GFP) spots were detected using the find maxima tool in ImageJ (Figure S2A and S2D). The regions of the Golgi stacks were segmented from the original fluorescence images by applying Gaussian blurring followed by binarization using an automatic thresholding method (Figure S2B and

S2C). The ERES spots whose peaks were positioned in the Golgi-stack regions were defined as Golgi-associated ERESs, and the other spots were defined as free ERESs (Figure S2D). Taking into account the possibility that ERES spots may overlap each other, the ratio of free and Golgi-associated ERESs was measured after correcting each number by the average signal intensity of the ERES peak.

For quantification of dynamic relationship of ERESs with Golgi stacks, frequency of ERES capture and release events by a Golgi stack in 20 s was manually counted from time-sequential images obtained at 100 ms intervals for 20 s.

Quantitative Analysis of Free ERESs on the ER Subdomains

Punctate ERESs and ER structures such as ER tubules and ER sheets were extracted from fluorescence microscopy images by semi-automatic image processing techniques consist of several morphological processing steps (Haralick et al., 1987). Details of the image processing protocol to quantify the topological relationship between punctate ERESs and ER are described in Figure S3.

The image processing procedure for extraction of the ER sheet structure is as follows: The original image was Gaussian-blurred [Figure S3C(2)] followed by contrast enhancement based on white top-hat transform through rotational morphological processing (RMP) [Figure S3C(3)]. This enhanced image was binarized by an automatic thresholding method [Figure S3C(4)]. The sheet regions were extracted by applying the opening procedure with RMP to the binarized image [Figure S3C(5)] followed by area opening operation to remove small isolated regions [Figure S3C(6)]. The ER body region was manually removed [Figure S3C(7)]. The ER sheet grooves were extracted by black top-hat transform through RMP [Figure S3C(8)] followed by binarization and area opening operation [Figure S3C(9)]. The ER sheet structure was extracted by subtracting the ER sheet grooves from the ER sheet regions [Figure S3C(10)].

The image processing procedure for extraction of the ER tubule region is as follows: The original image was Gaussian-blurred [Figure S3D(2)] followed by contrast enhancement based on white top-hat transform through RMP [Figure S3D(3)]. This enhanced image was binarized by an automatic thresholding method [Figure S3D(4)] followed by area opening operation [Figure S3D(5)]. The tubule regions were extracted by subtracting the ER sheet region from this binarized image [Figure S3D(6)] followed by area opening operation [Figure S3D(7)].

The image processing procedure for extraction of the punctate ERESs is as follows: The original image was Gaussian-blurred [Figure S3E(2)] followed by closing through RMP [Figure S3E(3)] and binarization by an automatic thresholding method [Figure S3E(4)]. The punctate ERESs were extracted from the original image [Figure S3E(1)] using the find maxima tool in ImageJ. The beaded rings of ERESs (Golgi-associated ERESs) were manually extracted [Figure S3E(5)], and the other punctate ERESs were defined as Golgi-free ERESs.

Motility Analysis

Trajectories of free ERESs, Golgi-associated ERESs, and Golgi stacks were manually extracted from time-sequential images captured at 100 ms intervals for 20 s using Manual Tracking plugin of ImageJ. Time-coded trajectories of the fluorescence particles were tracked from the center of each two-dimensional lattice.

Accession Numbers

Sequence data from this article can be found in The *Arabidopsis* Information Resource (TAIR) (http://www.arabidopsis.org) with the accession numbers as follows: *MAG5/SEC16A*, At5g47480; *SEC13A*, At3g01340; *SEC24A*, At3g07100; *SYP32*, At3g24350; *PICL*, At1g05320; *UBQ10*, At4g05320.

Supplemental References

Clough, S.J., and Bent, A.F. (1998). Floral dip: a simplified method for *Agrobacterium*-mediated transformation of *Arabidopsis thaliana*. Plant J. *16*, 735-743.

Geldner, N., Denervaud-Tendon, V., Hyman, D.L., Mayer, U., Stierhof, Y.D., and Chory, J. (2009). Rapid, combinatorial analysis of membrane compartments in intact plants with a multicolor marker set. Plant J. *59*, 169-178.

Haralick, R.M., Sternberg, S.R., and Zhuang, X. (1987). Image analysis using mathematical morphology. IEEE Trans. Pattern Anal. Mach. Intell. *9*, 532-550.

Karimi, M., Inze, D., and Depicker, A. (2002). GATEWAY vectors for *Agrobacterium*-mediated plant transformation. Trends Plant Sci. *7*, 193-195.

Nakamura, S., Mano, S., Tanaka, Y., Ohnishi, M., Nakamori, C., Araki, M., Niwa, T., Nishimura, M., Kaminaka, H., Nakagawa, T.*, et al.* (2010). Gateway binary vectors with the bialaphos resistance gene, *bar*, as a selection marker for plant transformation. Biosci. Biotechnol. Biochem. *74*, 1315-1319.

Nakano, R.T., Matsushima, R., Ueda, H., Tamura, K., Shimada, T., Li, L., Hayashi, Y., Kondo, M., Nishimura, M., and Hara-Nishimura, I. (2009). GNOM-LIKE1/ERMO1 and SEC24a/ERMO2 are required for maintenance of endoplasmic reticulum morphology in *Arabidopsis thaliana*. Plant Cell *21*, 3672-3685.

Nelson, B.K., Cai, X., and Nebenfuhr, A. (2007). A multicolored set of *in vivo* organelle markers for co-localization studies in Arabidopsis and other plants. Plant J. *51*, 1126-1136.

Schindelin, J., Arganda-Carreras, I., Frise, E., Kaynig, V., Longair, M., Pietzsch, T., Preibisch, S., Rueden, C., Saalfeld, S., Schmid, B.*, et al.* (2012). Fiji: an open-source platform for biological-image analysis. Nat. Methods *9*, 676-682.

Segami, S., Tomoyama, T., Sakamoto, S., Gunji, S., Fukuda, M., Kinoshita, S., Mitsuda, N., Ferjani, A., and Maeshima, M. (2018). Vacuolar H(+)-Pyrophosphatase and Cytosolic Soluble Pyrophosphatases Cooperatively Regulate Pyrophosphate Levels in *Arabidopsis thaliana*. Plant Cell *30*, 1040-1061.

Shaner, N.C., Campbell, R.E., Steinbach, P.A., Giepmans, B.N., Palmer, A.E., and Tsien, R.Y. (2004). Improved monomeric red, orange and yellow fluorescent proteins derived from *Discosoma* sp. red fluorescent protein. Nat. Biotechnol. *22*, 1567-1572.

Takagi, J., Renna, L., Takahashi, H., Koumoto, Y., Tamura, K., Stefano, G., Fukao,

Y., Kondo, M., Nishimura, M., Shimada, T., *et al.* (2013). MAIGO5 functions in protein export from Golgi-associated endoplasmic reticulum exit sites in *Arabidopsis*. Plant Cell *25*, 4658-4675.
